# Responding to chromosomal breakage during M-phase: insights from a cell-free system

**DOI:** 10.1186/1747-1028-4-15

**Published:** 2009-07-14

**Authors:** Eloise Smith, Vincenzo Costanzo

**Affiliations:** 1 Genome Stability Unit, London Research Institute, Clare Hall Laboratories, South Mimms, London, UK, EN6 3LD

## Abstract

DNA double strand breaks (DSBs) activate ATM and ATR dependent checkpoints that prevent the onset of mitosis. However, how cells react to DSBs occurring when they are already in mitosis is poorly understood. The *Xenopus *egg extract has been utilized to study cell cycle progression and DNA damage checkpoints. Recently this system has been successfully used to uncover an ATM and ATR dependent checkpoint affecting centrosome driven spindle assembly. These studies have led to the identification of XCEP63 as major target of this pathway. XCEP63 is a coiled-coil rich protein localized at centrosome essential for proper spindle assembly. ATM and ATR directly phosphorylate XCEP63 on serine 560 inducing its delocalization from centrosome, which in turn delays spindle assembly. This pathway might contribute to regulate DNA repair or mitotic cell survival in the presence of chromosome breakage.

## Introduction

DSBs are dangerous DNA lesions as they can lead to massive loss of genetic information and to chromosomal rearrangements. In order to preserve genomic stability, eukaryotic cells have evolved systems to sense DSBs and promote responses to ensure that large pieces of the genome are not "lost". These signaling pathways can modulate cell cycle progression to support the repair of DSBs. Cell cycle arrest as a consequence of DNA damage signaling is known as 'checkpoint response'. The study of this response has gained important insights via the exploitation of *in vitro *systems. Amongst these the *Xenopus laevis *egg extract has facilitated the study of the biochemical mechanisms behind cellular responses to DSBs during the cell cycle. *Xenopus *egg cytoplasm undergoes rapid and spontaneous oscillation of Cyclin dependent kinase (Cdk) activity driving the extract into consecutive rounds of cell cycle [1]. For these properties egg extract constitutes a powerful tool for the *in vitro *study of both DNA replication and mitosis [2-4]. Recently, the *Xenopus *egg extract has been utilized to study cell cycle checkpoints in response to DNA damage and replication stress. These studies have taken advantage of the possibility to use DNA structures that mimic damaged DNA in order to examine subsequent progression into S and M phase. DNA containing double strand breaks (DSBs) can be used to activate ATM [5]. ATR instead can be activated by DNA structures made of poly-deoxy-T annealed to poly-deoxy-A oligonucleotides, which can anneal in a staggered fashion producing DNA gaps [6]. Here we have highlighted recent observations made with the *Xenopus *system regarding the response to DSBs arising during mitosis.

## Responding to DSBs in mitosis

The presence of DSBs activates an ATM and ATR dependent checkpoint that prevents the onset of mitosis [7]. However, cells can adapt to the presence of unrepaired chromosomal breakages overcoming the G2/M checkpoint and subsequently entering mitosis [8]. This adaptation pathway was believed to operate only in unicellular eukaryotes. However, this phenomenon has been demonstrated in higher pluricellular organisms [9]. Furthermore, checkpoint proficient cells with defects in DSB repair enter mitosis with a high number of unrepaired DSBs following treatment with ionizing radiation [10]. This is probably due to the low sensitivity of the G2/M checkpoint [11]. How cells react to DSBs in mitosis is poorly understood. Attempts to create selective, laser-made chromosome breaks in mitotic mammalian cells have shown that unless the breakage occurs directly in the kinetochore region of the mitotic chromosome, there is no ATM and/or ATR dependent DNA damage checkpoint response that would inhibit mitosis progression [12]. These studies led to the conclusion that mitosis progression can only be affected by kinetochore damage activating the spindle assembly checkpoint (SAC). However, Chk1 dependent slow down of mitotic progression has been observed in Drosophila cells following induction of DSBs away from the centromere region [13]. Moreover ATM and ATR activation of the SAC independently of the kinetochore status has been shown in yeast cells [14]. These studies suggest that ATM and ATR dependent pathways impact on mitosis progression. An additional link to these findings comes from the recent survey of ATM and ATR targets amongst which essential components of the SAC were found [15]. Mitotic fate in the presence of DNA damage could be more complex and stochastic that previously believed. Many of the studies analyzing the effects on mitosis of spindle poisoning or DNA damage inducing drugs have been performed using microscopy-based techniques monitoring the behavior of few cells or applying population-based approaches. Recently, using automated time-lapse light microscopy Taylor and colleagues established single-cell based assays analyzing over 10.000 cells at the time. This study led to the discovery that mitotic cells challenged with different drugs display complex behavior going from arrest, to endoreduplication of the genome, and cell death [16]. Surprisingly, in addition to the marked difference amongst different cell lines individual cells of any given line seems to display a variety of different fates that escape detection using indirect methods such as flow-cytometry and western blotting techniques [16]. This unexpected heterogeneous response of mitotic cells demands a revision of previous studies to better understand the effects of cellular stress in mitosis. A major problem in the detection of mitotic progression defects in the presence of chromosomal breakage is intrinsic to the short duration of this cell cycle stage. Induction of chromosomal breakage in cells synchronized in mitosis with microtubule depolymerising agents is usually the approach used to study the effects of DNA damage in mitosis. However, the results obtained with this protocol might be influenced by the procedures used to synchronize cells in mitosis. An alternative approach to study the effects of DNA damage in mitosis is to exploit synchronized cell free systems. Extracts obtained from meiotic eggs of *Xenopus laevis *are naturally arrested in mitosis by the Cytostatic Factor (CSF) [17]. CSF arrested mitotic egg extracts are capable of assembling spindle structures following the addition of sperm nuclei. The addition of sperm nuclei promotes the formation of aster/centrosome structures that nucleate spindle microtubules [18]. In this system the possibility of activating the ATM and ATR dependent DNA damage response to study the effects of this signalling pathway on spindle assembly could be exploited. The DNA damage response to DSBs can be easily activated in egg extract by addition of linear DNA fragments or induction of chromosomal breakage [19,20]. Using this system it was shown that activation of the ATM and ATR dependent DNA damage response abolishes spindle assembly in mitotic extract [21]. Importantly, spindle defects were fully reversible by ATM and ATR inhibitors. This ruled out that physical damage to the kinetochore was responsible for the observed spindle defects.

To determine the mechanism leading to inhibition of spindle assembly Cdk1/Cyclin B and Plx1 activities were monitored. Cdk1/Cyclin B and Plk1 are both targets of the checkpoint inhibiting mitosis entry [22] and are essential for spindle assembly [18]. However, chromosomal breakage did not affect Cdk1/Cyclin B and Plx1 activities in extracts that were already in mitosis although ATM and ATR were fully activated [21]. It is possible that downstream signalling events in the ATM and ATR dependent pathway normally leading to G2/M arrest are inhibited in cells that are already in mitosis. This is consistent with the fact that some of the DNA damage signalling pathways functioning in S and G2 phase are attenuated in mitosis [23,24]. It is likely that the activities of Cdk1/CyclinB and Plx1, which depend on a robust Cdc25 dependent amplification loop, are refractory to the DNA damage signalling once at their peak in mitosis. The mechanisms underlying the resistance of mitotic kinases to ATM and ATR in mitotic extracts remains to be established. However, it should be mentioned that this might reflect a physiological mechanism that would prevent premature inactivation of essential kinases required to maintain the mitotic status and therefore suppress mitosis exit in the presence of damaged chromosomes. To uncover the major spindle assembly pathway affected by ATM and ATR formation of anastral spindles was monitored. These spindles can be induced by chromatin-coated beads [25]. The chromatin beads promoting anastral spindle assembly consist of linear DNA molecules and for this reason are capable of inducing the DNA damage response in egg extract. In this case, differently from sperm nuclei induced spindles, the DNA damage response does not impair anastral spindle formation [21]. Sperms carry an intact centriole, which give rise to a functional aster/centrosome structure. The presence of a centriole/centrosome acts as a dominant center of microtubule organizations. The fact that the DNA damage response only affects the formation of spindle triggered by sperm nuclei suggested that the target of ATM and ATR was linked to the aster/centrosome dependent spindle assembly pathway. To identify ATM and ATR targets involved in this process a screening based on a cDNA expression library was developed [26]. Pools of Xenopus cDNAs translated and ^35^S-labelled in reticulocyte lysates were mixed with extracts in the presence of an active DNA damage response. Slower migrating forms of ^35^S-labelled proteins resulting from direct phosphorylation by active ATM and ATR or by other DSB activated kinases were then isolated. Fewer ATM and ATR targets than expected were isolated and among these only the Xenopus ortholog of CEP63 [27] (XCEP63) was a centrosomal protein. As ATM and ATR only affect centrosome driven spindle assembly in egg extract XCEP63 was chosen as candidate target to explain the effects of ATM and ATR on spindle assembly. XCEP63 is a coiled-coil rich protein with an SMC (Structural Maintenance of Chromosome) domain. Little was known about XCEP63 except the fact that is localized at centrosomes. ATM and ATR directly phosphorylate XCEP63 on serine 560. It is still unclear how nuclear proteins such as ATM and ATR phosphorylate a centrosomal target. However, a variety of non-nuclear proteins have been shown to be directly phosphorylated by ATM and ATR [15]. The phosphorylation of XCEP63 on serine 560 has a profound effect on the localization of the protein as it induces its delocalization from the centrosome. The molecular mechanism behind this event is not yet known. It is possible that the phosphorylation of serine 560 decreases the affinity of the protein for a centrosomal partner. Alternatively, as many centrosomal proteins are actively transported by motor proteins the phosphorylation of serine 560 might affect its active transport towards the centrosome.

XCEP63 is essential for proper spindle assembly. Depletion of XCEP63 inhibits spindle formation. In the absence of XCEP63 DNA associated microtubule aggregates can be observed suggesting that XCEP63 coordinates centrosome dependent microtubule assembly into spindle structures [21].

Phosphorylation of XCEP63 on serine 560 could lead to functional inactivation of XECP63. Consistent with this hypothesis replacement of endogenous XCEP63 with recombinant XCEP63 that could not be phosphorylated by ATM and ATR restored spindle formation in the presence of chromosomal breakage. These results strongly establish XCEP63 as major ATM and ATR target in the checkpoint that inactivates spindle assembly following chromosomal breakage. These findings were in part supported by observations made in chicken DT40 cells in which CEP63 gene was inactivated. Chicken CEP63 is also phosphorylated in an ATM and ATR dependent manner although serine 560 is not conserved. This indicates that the control of CEP63's function in the presence of DNA damage is conserved in other vertebrates even in the absence of a conserved phosphorylation site. CEP63's function, like many other centrosome proteins, is not known at biochemical level. Assays to reveal its role in the centrosome and spindle assembly will need to be developed to understand how CEP63 controls this process. Preliminary evidence showing interaction of human CEP63 with DISC1 points at a role for this protein in spindle pole formation [28].

## Conclusion

**Figure 1:**
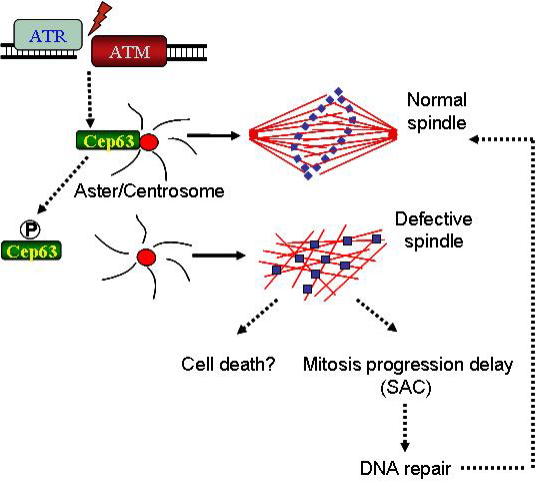
**Proposed model: ATM and ATR mediated XCEP63 phosphorylation promotes its removal from centrosome delaying spindle assembly**. Depending on the number of DNA breaks this can lead to mitotic progression delay or mitotic cell death.

Regulation of spindle assembly through a centrosomal protein reflects the central role of the centrosome in mitotic events in the presence of DNA damage. Consistent with these findings, pathways leading to centrosome inactivation following DNA damage have been shown to operate in other organisms [29,30]. Inhibition of spindle assembly following chromosomal breakage may be crucial when other mechanisms preventing mitosis entry have failed. Chromosomal breakage may induce mitotic progression delay and prevent chromosome segregation through indirect activation of the SAC, following ATM and ATR dependent inhibition of spindle assembly (Figure 1). This mechanism may allow repair of chromosome breakage, facilitated by chromatin remodelling and de-condensation at the breakage site without inducing down-regulation of mitotic kinases such Cdk1 or Plk1, and therefore mitosis exit. When DNA damage is un-repairable ATM and ATR dependent spindle inactivation could instead lead to mitotic catastrophe preventing survival of cells with extensively damaged chromosomes (Figure 1). Cancer cells might evade this checkpoint by altering the structure and the function of centrosomes. Interestingly, the expression of human CEP63 is altered in aggressive bladder tumors [31]. Defects in the centrosome structure and function as the ones found in solid tumors might allow escape from this DNA damage checkpoint and ensure survival of mitotic cells even in the presence of chromosomal breakage at the expense of genome stability.

## Competing interests

The authors declare that they have no competing interests.

## Authors' contributions

ES and VC co-wrote and edited the manuscript. Both authors read and approved the final manuscript.
